# Fusion of Unmanned Aerial Vehicle Panchromatic and Hyperspectral Images Combining Joint Skewness-Kurtosis Figures and a Non-Subsampled Contourlet Transform

**DOI:** 10.3390/s18103467

**Published:** 2018-10-15

**Authors:** Jinling Zhao, Chengquan Zhou, Linsheng Huang, Xiaodong Yang, Bo Xu, Dong Liang

**Affiliations:** 1 National Joint Engineering Research Center for Analysis and Application of Agro-Ecological Big Data, Anhui University, Hefei 230601, China; zhaojl@ahu.edu.cn (J.Z.); linsheng0808@163.com (L.H.); 2 National Engineering Research Center for Information Technology in Agriculture, Beijing 100089, China; yangxd@nercita.org.cn (X.Y.); xub@nercita.org.cn (B.X.)

**Keywords:** image fusion, non-subsampled contourlet transform (NSCT), joint skewness-kurtosis figure (JSKF), IHS transform, remote sensing

## Abstract

To obtain fine and potential features, a highly informative fused image created by merging multiple images is usually required. In our study, a novel fusion algorithm called JSKF-NSCT is proposed for fusing panchromatic (PAN) and hyperspectral (HS) images by combining the joint skewness-kurtosis figure (JSKF) and the non-subsampled contourlet transform (NSCT). The JSKF model is used first to derive the three most sensitive bands from the original HS image according to the product of the skewness and the kurtosis coefficients of each band. Afterwards, an intensity-hue-saturation (IHS) transform is used to obtain the luminance component *I* of the produced false-colour image consisting of the above three bands. Then the NSCT method is used to decompose component *I* of the false-colour image and the PAN image. The weight-selection rule based on the regional energy is adopted to acquire the low-frequency coefficients and the correlation between the central pixel and its surrounding pixels is used to select the high-frequency coefficients. Finally, the fused image is obtained by applying an IHS inverse transform and an inverse NSCT transform. The unmanned aerial vehicle (UAV) HS and PAN images under low- and high-vegetation coverage of wheat at the flag leaf stage (Stage I) and the grain filling stage (Stage II) are used as the sample data sources. The fusion results are comparatively validated using spatial (entropy, standard deviation, average gradient and mean) and spectral (normalised difference vegetation, NDVI, and leaf area index, LAI) assessments. Additional comparative studies using anomaly detection and pixel clustering also demonstrate that the proposed method outperforms other methods. They show that the algorithm reported herein can better preserve the original spatial and spectral characteristics of the two types of images to be fused and is more stable than IHS, principal components analysis (PCA), non-negative matrix factorization (NMF) and Gram-Schmidt (GS).

## 1. Introduction

With the development of remote-sensing technology, hyperspectral (HS) remote sensing has been widely used in precision agriculture, mineral exploration, land-use/land-cover classification, and other fields [1,2,3,4,5]. More recently, HS imaging by acquiring a scene in several hundreds of contiguous spectral bands has opened a new range of relevant applications such as target detection and spectral unmixing [6,7]. However, while HS sensors provide abundant spectral information, their spatial resolution is generally more limited. To obtain images with good spectral and spatial resolutions, the remote sensing community has devoted increasing research efforts to the problem of fusing HS images with panchromatic (PAN) images [8,9,10]. The fusion of HS images with panchromatic ones of high spatial resolution can not only provide HS images with the excellent spatial characteristics of PAN images but also provide PAN images with rich spectral information. This is an effective way to improve the observability of the two types of images, which is beneficial to the subsequent processing of the visual interpretation, and is therefore of great practical value. At present, all types of fusion algorithms aim to improve the quality of fused images and reduce the fusion time. In selecting fusion algorithms, full attention should be given to the specific features of HS images, such as large amounts of data, strong correlation between bands, and specific fusion requirements.

In general, the most common fusion algorithms can be separated into three categories. The most classical are the projection- and substitution-based methods, which assume that the PAN image [11] is equivalent to the structural component of the intensity-hue-saturation (IHS) when the latter is translated into a new space. Algorithms such as the IHS [12], the principal component substitution (PCS) [13], and the Gram–Schmidt (GS) transformation [14] all belong in this category. They provide visually superior high-resolution HS images, but ignore the requirement for high-quality synthesis of spectral information.

Another type of methods are those based on band ratios and arithmetic combinations. An example of this is the synthetic variable ratio [15], which is rapid and performs well. The last category is the wavelet-based methods, such as the discrete wavelet transform [16], which extracts the high frequencies of the PAN image and then injects them into the IHS space to get the fused image. In addition, recent years have seen the emergence of many other algorithms that improve upon existing methods, such as the fast Fourier transform [17] and enhanced IHS [18,19].

At present, the fusion results of these methods lose some of original image information while enhancing spatial or spectral information. In addition, most of these algorithms do not consider the physical characteristics of the detection system (i.e., each sensor works in different regions of the electromagnetic spectrum). Ignoring this fact leads to injection of spectral information from different parts of the spectrum which may not belong to the sensor, which leads to modification of spectral signatures in the fused HS data. In addition to general evaluation indices such as entropy, mean, mean square error (MSE), to evaluate and compare the results of fusion methods, some approaches or algorithms are also proposed under different application landscapes. For example, the so called Wald’s protocol is normally based on a high-resolution HS image in the pansharpening community [20]. Conversely, when there are no ground truth high-resolution HS images, the quality without reference (QNR) algorithm and generalized QNR (GQNR) are recommended [21,22].

Unmanned Aerial Vehicles (UAVs), as efficient and a highly flexible aircraft, have been widely used to collect multisource high-resolution airborne remote sensing imagery. Several sensors can be simultaneously aboard an UAV to acquire different remote sensing data. The primary issue is how to utilize the multisource images to accurately identify the targets. It is of great significance to acquire a highly informative fused image created by merging multiple images. A novel fusion algorithm (hereafter referred to as “JSKF-NSCT”) was proposed in our study to merge the UAV PAN and HS images of wheat at two growth stages. Our highlights for this analysis are: (1) joint skewness-kurtosis figure (JSKF) is used to assist in finding out three most sensitive spectral bands from original HS image; (2) non-sampling contourlet transform (NSCT) is then used to decompose the high- and low-frequency fusion coefficients; and (3) the weight-selection rule based on the regional energy is adopted to assure the low-frequency coefficients and the correlation between the central pixel and its surrounding pixels is used to select the high-frequency coefficients.

## 2. Materials and Methods

### 2.1. Study Area and Experimental Design

**Figure 1:**
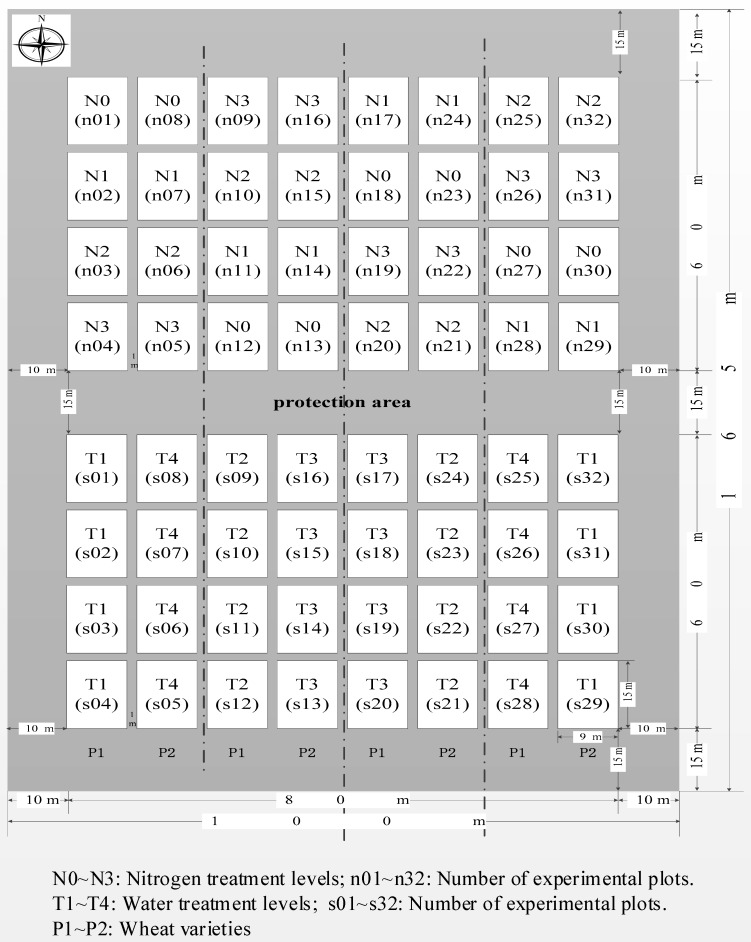
The schematic plot of our experimental design.

The experimental site is located at Xiaotangshan National Precision Agriculture Research and Demonstration Base, which is located in the Changping District (Beijing, China) at a latitude of 40°00′ N–40°21′ N, a longitude of 116°34′ E–117°00′ E, and an altitude of 36 m. The length of the experimental site in the east–west direction was 100 m and the length in the north–south direction was 165 m. The site contained 64 plots, each measuring 9 m × 15 m (Figure 1).

The experimental equipment included an eight-rotor UAV (Dajiang, Shenzhen, China); a UHD-185 Airborne HS camera (Cubert GmbH, Ulm, Baden-Württemberg, Germany) with a spectral range of 450–950 nm, a sampling interval of 4 nm, a spectral resolution of 8 nm at 532 nm, and 125 spectral channels; and an HD digital camera (DSC-QX100, Sony, Tokyo, Japan, resolution of 5472 × 3648). The data used in this work include HS and digital photographs of two stages of wheat growth. Samplings took place on 21 April 2015 (flag leaf stage, Stage I) and 22 May 2015 (grain filling stage, Stage II).

### 2.2. Our Proposed Fusion Algorithm

**Figure 2:**
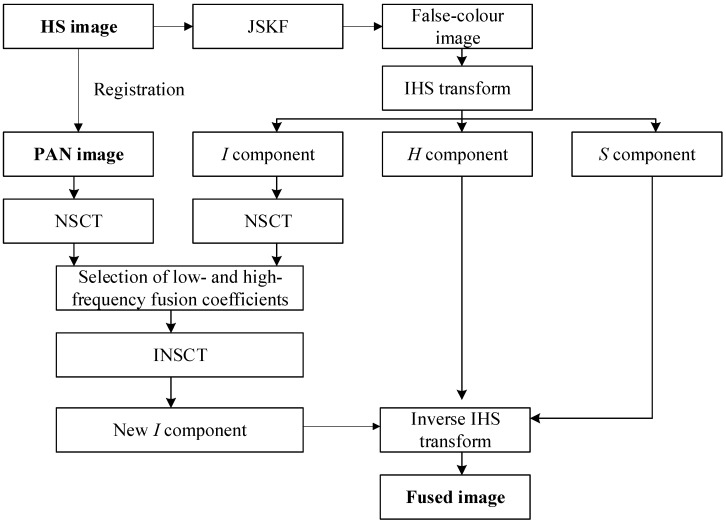
The overall technical flowchart of our proposed fusion algorithm.

Three primary steps are required to finish the fusion process from original HS and PAN images to fused image (Figure 2). The first is to preprocess and register the HS and PAN images. The number of original spectral bands of HS image is reduced by the JSKF model, and three most sensitive bands are selected to generate the false-colour image. The second is to carry out the IHS transform for obtaining the three components. NSCT is used to decompose the low- and high-frequency fusion coefficients. The third is to select the fusion rules for high- and low-frequency coefficients. A new *I* component can be produced by the inverse NSCT and the fused image can be generated by the inverse IHS.

#### 2.2.1. Selection of Sensitive Bands Based on the JSKF Model

Because of the corresponding large volume of data, HS images are very difficult to transmit and store. Resolving this problem requires a technique to reduce the dimensions of HS images [23,24]. Because of the strong correlation and high redundancy of the HS bands, applying the dimensional-reduction method allows the data volume to be compressed. The number of bands selected depends on the actual needs. In this paper, HS images and PAN images are used to fuse high-quality false-colour images. Colour images are composed of three components: red (R), green (G), blue (B), so three bands are selected from HS images for fusion with PAN images. Several guidelines are available for selecting the sensitive bands:

(1) From the point of view of information theory [25], the maximum amount of information must be selected from the band or band combination.
(2) From the point of view of mathematical statistics, the correlation between the selected bands should be weak so as to maintain the independence and effectiveness of each band.
(3) From the point of view of spectroscopy, the differences in the spectral characteristics of the properties to be identified from the study area should be maximized.
(4) From the point of view of classification, objects that need to be discriminated should be most strongly classified in the selected bands.

There are two aspects of the current group of band-selection techniques: the criterion function and the search method. The main challenge of HS image dimension reduction is to reduce the volume of data while keeping sufficient information for the following informational analysis of the image. Up to now, many types of methods have been proposed to reduce the large quantity of HS data, such as band-selection-based methods, subspace decomposition-based methods, and feature-detection-based methods. Traditional band-selection methods based on information quantity are to use the entropy, joint entropy, and variance of the image as the measurement indices of information size [26] and select the bands with the maximum information. Nevertheless, the methods are generally identified that the quality of images is measured as a whole without considering the spatial features of images and the statistical or distribution characteristics of objects or subjects.

For HS images, according to the central-limit theorem, the background samples, which contain most of the information in an image, follow an approximate Gaussian distribution [23]. The target can be regarded as an exception in the image (as opposed to the background). Thus, finding the non-background features such as the target can be reduced to searching for the characteristics of a Gaussian distribution. Therefore, the skewness and kurtosis coefficients can be used to measure the amount of feature information, such as the target and the size of the feature.

To measure the degree of deviation from the normal distribution more comprehensively and effectively, we use the product of the skewness and the kurtosis coefficients as an index to measure the amount of information that deviates from the normal distribution, that is, the JSKF [6]:

(1) JSKF=S·K

Let *x*_1_, *x*_2_, …, *x_n_* be a sample from the general *x*. Then the discrete form for skewness is:

(2) $$\hat{S}=\frac{1}{n}{\sum_{i=1}^{n}\left(x_{i}-\bar{x}\right)}^{3}/{\left(\frac{1}{n}\sum_{i=1}^{n}{\left(x_{i}-\bar{x}\right)}^{2}\right)}^{3/2}$$

where $\bar{x}=E(x)=\sum_{i=1}^{n}x_{i}$ represents the sample mean. Thus, the discrete formula for kurtosis is:

(3) $$\hat{K}=\frac{1}{n}{\sum_{i=1}^{n}\left(x_{i}-\bar{x}\right)}^{4}/{\left(\frac{1}{n}{\sum_{i=1}^{n}\left(x_{i}-\bar{x}\right)}^{2}\right)}^{2}-3$$

For convenience, the division of the discrete form can be converted into multiplication, which gives the discrete formula for calculating the JSKF as:

(4) $$\begin{array}{ll} \mathrm{JSKF} & =\hat{S}\cdot \hat{K}\\ & =\left[\frac{1}{n}\sum_{i=1}^{n}{\left(x_{i}-\bar{x}\right)}^{3}{\left(\frac{1}{n}\sum_{i=1}^{n}{\left(x_{i}-\bar{x}\right)}^{2}\right)}^{2/3}\right]\cdot \left[\frac{1}{n}\sum_{i=1}^{n}{\left(x_{i}-\bar{x}\right)}^{4}{\left(\frac{1}{n}\sum_{i=1}^{n}{\left(x_{i}-\bar{x}\right)}^{2}\right)}^{1/2}-3\right] \end{array}$$

**Figure 3:**
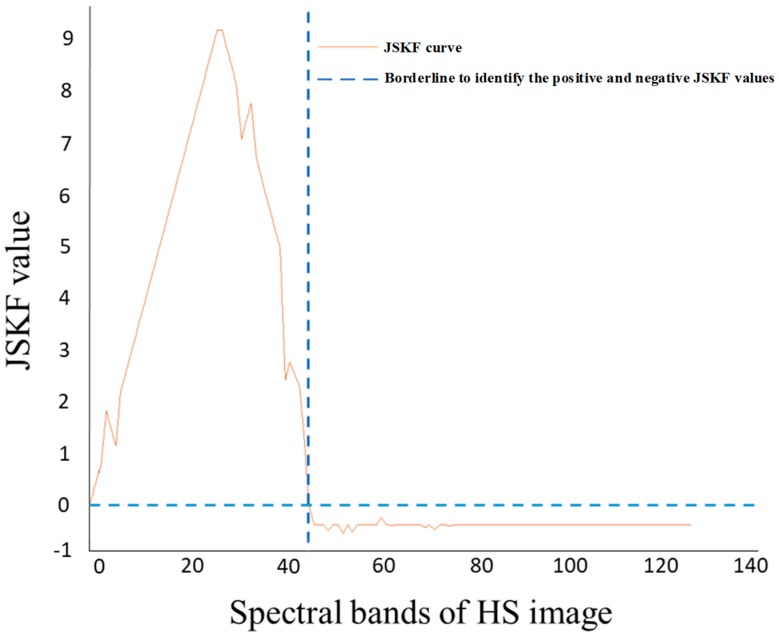
The JSKF curve of the original HS image.

In this paper, the product of the skewness and kurtosis coefficients is used as the coefficient of band selection. According to the sign of the coefficient, we first partition the adaptive space and then select the optimal bands according to the absolute value. Based on the definition of JSKF, we see that a larger JSKF value corresponds to a greater deviation of the data from the normal distribution. The data thus contain more information of interest to us. The positive and negative coefficients reflect the difference within the data distribution. Therefore, we can calculate the JSKF of the HS image data. The original image data space is divided into two subspaces according to the difference in the distribution. The images in these two subspaces have high similarity in their respective subspaces, but the similarity between the two subspaces is low and the difference is large. Figure 3 shows the JSKF curves of HS images with 128 bands. This result shows that the 128 bands can be divided into two subspaces as a function of the sign of the JSKF.

The HS image bands can be further classified according to the absolute value of the JSKF in each subspace, which finally allows us to choose the best bands in the divided subspace. The method is described as follows:

(1) Set the threshold to automatically select the bands.
(2) Sort the JSKF according to its absolute value and then according to the requirements of later processing to select the *n* largest bands.

In addition, the random distribution of the additive noise in the image is generally similar to a Gaussian distribution. The effective information in the image made of the contaminated band is suppressed by the presence of noise, which makes the grey-level distribution of the noise band tend to a Gaussian distribution. Therefore, the band’s JSKF suffers less from noise pollution. By using this method, the band-selection method can efficiently remove the noise, which is helpful for selecting bands that give better image quality.

#### 2.2.2. NSCT Based Fusion of PAN and HS Images

As we know, approximation of an image belongs to the low-frequency part, while the high-frequency counterpart exhibits detailed features of edge and texture. In this paper, the NSCT method is used to separate the low and high components of the source image in the frequency domain, and then the two parts are dealt with by different fusion rules according to their features. As a result, the fused image is more complementary, reliable, clear, and better understood.

**Figure 4:**
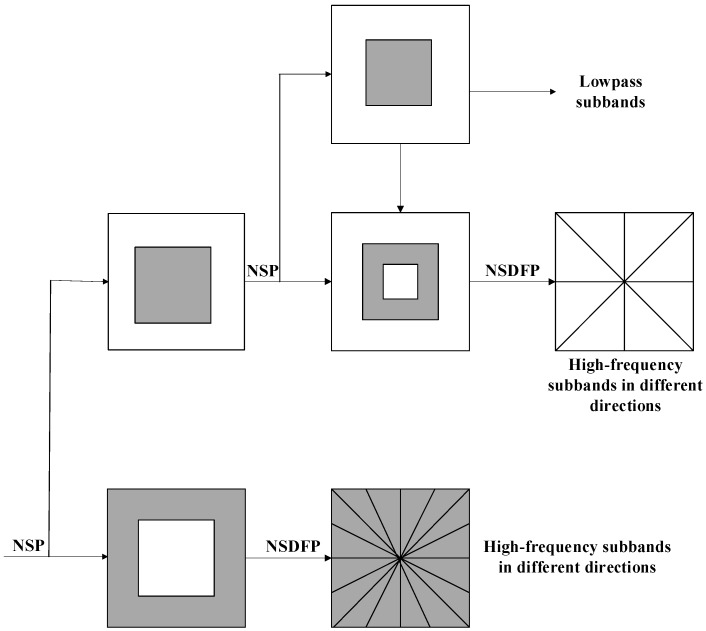
The decomposition flowchart of the NSCT.

The NSCT is a sparse representation of the image that has not only multiple scales and time-frequency localization but also a high degree of directionality and anisotropy [27]. The basic idea of this method is to decompose the image into a pyramid multiscale decomposition and then use the direction filter bank to decompose the subband images. In this way, we get the subbands with different scales and directions, and the method allows for a different number of directional subbands on each scale. This algorithm is similar to the translation algorithm in translation-invariant wavelet transform. In the process of decomposition, the decomposed subbands have the same size as the original image, which makes the NSCT translation invariant. Figure 4 depicts the NSCT image-decomposition process. In this figure, “NSP” refers to “non-subsampled pyramid” and “NSDFP” refers to “non-subsampled directional filter pyramid”.

Similar to the Laplacian pyramid algorithm, NSCT first uses the original HS images and the non-sampling two-channel filter bank to perform the convolution to obtain an image-decomposition layer. However, we do not sample the decomposed low-frequency image except for the interpolation of the non-subsampled double-channel filters [28]. When it goes down and we get multiple resolutions of the image. This type of non-sampling decomposition prevents aliasing of the frequency spectrum of each subband [29], which is very valuable for image fusion. After the image is decomposed by layer N, the (*n* + 1)-th subband is obtained in the pyramid, including the N-detail subband and one low-resolution approximation subband.

**Figure 5:**
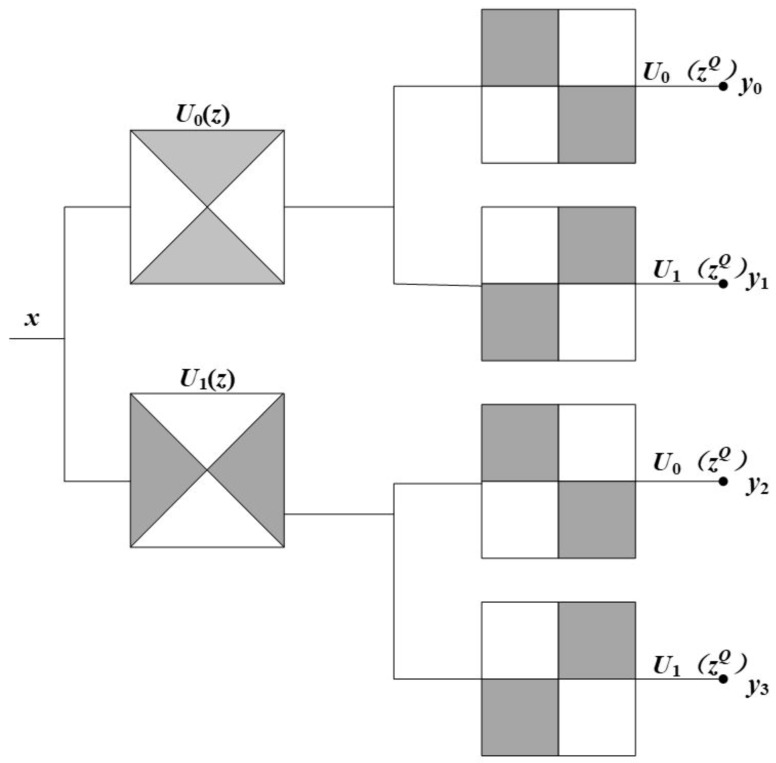
Schematic diagram of NSCT directional filter banks.

The non-directional filter bank of NSCT is a type of sector directional filter bank that does not take up and down sampling instead of corresponding to the filter group in the direction of interpolation processing. Thus, the nondirectional filter banks with translation invariance are obtained and divide the two-dimensional frequency-domain plane into the direction wedge block [30], each representing the image detail feature in this direction. Figure 5 gives a schematic diagram of the decomposition of the filter banks.

Because the NSCT can overcome the aliasing phenomenon, it has better spectral characteristics. This paper is based on this method, and the basic processes of the fusion method are as given in the following algorithm (Table 1):

### 2.3. Procedures to Select Low- and High-Frequency Coefficients

(1) Transform the three selected bands of the HS image from RGB space to IHS space using a linear IHS transform. The positive and negative transformation formulas are:

(5) $$\left[\begin{array}{c} I\\ n_{1}\\ n_{2} \end{array}\right]=\left[\begin{array}{ccc} 1/3 & 1/3 & 1/3\\ -\sqrt{2}/6 & -\sqrt{2}/6 & \sqrt{2}/6\\ 1/\sqrt{2} & -1/\sqrt{2} & 0 \end{array}\right]\left[\begin{array}{c} R\\ G\\ B \end{array}\right]$$

(6) $$\left[\begin{array}{c} R\\ G\\ B \end{array}\right]=\left[\begin{array}{ccc} 1 & -1/\sqrt{2} & 1/\sqrt{2}\\ 1 & -1/\sqrt{2} & -1/\sqrt{2}\\ 1 & \sqrt{2} & 0 \end{array}\right]\left[\begin{array}{c} I\\ \nu_{1}\\ \nu_{2} \end{array}\right]$$

Among them, hue and saturation can be calculated by the following equation:

(7) $$\{\begin{array}{c} H=\arctan(\frac{\nu_{1}}{\nu_{2}})\\ S=\sqrt{\nu_{1}^{2}+\nu_{2}^{2}} \end{array}$$

(2) Match the histogram of the high-resolution PAN image and the component *I* of the HS image obtained by the IHS transform. Apply the NSCT transform to the PAN image and component *I*. Next, we obtain an approximate low-frequency component of the image and the high-frequency subbands in the multilayer and in multiple directions.

(3) Select low- and high-frequency fusion coefficients

(i) Fusion rules for low-frequency coefficients

The low-frequency subbands obtained by the decomposition of the NSCT reflect the image approximation and average characteristics [31] and construct the basic outline of the image. The traditional method of averaging coefficients is not suitable for the fusion of HS and PAN images. Because of the high resolution of the PAN image, the average-value method will weaken the contour information of the PAN image. Therefore, the correct selection of low-frequency subband coefficients can improve the visual effect of the image. To better integrate the features of the high-resolution image into the HS image, this paper adopts the weight-selection rule based on the regional energy [32]. In this paper, we automatically determine the weight according to the size of the regional energy. We denote the low-frequency part of the PAN image as *I_A_* and the low-frequency component of the HS image as *I_B_*. Then the low-frequency component *I_L_* is:

(8) $$I_{L}(x,y)=\omega_{A}I_{A}(x,y)+\omega_{B}I_{B}(x,y)$$

where $\omega_{A}$ and $\omega_{B}$ represent the corresponding pixel-weight coefficients and they can be calculated as follows:

(9) $$\{\begin{array}{c} \omega_{A}=\frac{E_{A}(x,y)}{E_{A}(x,y)+E_{B}(x,y)}\\ \omega_{B}=\frac{E_{B}(x,y)}{E_{A}(x,y)+E_{B}(x,y)} \end{array}$$

where *E_A_*(*x, y*) and *E_B_*(*x, y*) represent the region energy of the low-frequency subband. In this study, the selection range of the energy is a 3 by 3 pixel sized window, which is centered on the fused pixel.

(ii) High-frequency fusion coefficients

The high-frequency coefficients are the details of the image, such as edge information and texture information [33]. This information is the focus of the human visual system. The purpose of high-frequency-coefficient fusion is to extract the maximum amount of detailed information from the source image. In the original HS image, those obvious image features, such as lines, curves, edges, and so on, are often shown as the grey value and its change. In the multiscale transform domain, it is often manifested as a high-frequency subband transform coefficient with higher modulus. However, the study of physiology shows that the human eye is sensitive to the local contrast of the image but not to the brightness of a single point [34]. Thus, high-frequency fusion should aim to better highlight the local contrast of an image and consider the correlation between the central pixel and its surrounding pixels. The gradient of the eight directions of the high-frequency coefficients and their domain coefficients is used as the threshold for pixel selection. Denoting the high-frequency coefficients of each layer and direction of the image after fusion as *I*, the formula is:

(10) $$I_{j,k}(x,y)=\{\begin{array}{c} I_{j,k}^{A}(x,y),T_{A}(x,y)\geq T_{B}(x,y);\\ I_{j,k}^{B}(x,y),T_{A}(x,y)\sum_{i=1}^{n}X_{i}<T_{B}(x,y) \end{array}$$

where *T*(*x*, *y*) is shown in Equation (11) and it represents the total variation between the high-frequency coefficient and its corresponding neighborhood coefficients in eight gradient directions.

(11) $$T(x,y)=\sum_{m=-1}^{1}\sum_{n=-1}^{1}\left|I_{i,j}(x,y)-I_{i,j}(x+m,y+n)\right|$$

(4) The inverse NSCT is obtained by the procedure above for the low- and high-frequency coefficients of the new fused image. Then, we receive the intensity component *I* of the fused image, following which the new *I* component and the *H* and *S* components are transformed into RGB space [35]. After that, we get the fused high-resolution images.

### 2.4. Performance Metrics for Evaluating the Fusion Results

(1) Mean

Mean is the average of all pixels in the image. It approximately reflects the grey distribution of the image [36] and is the most direct reflection of the image brightness. If the mean value is moderate, the visual effect is good. The mean *μ* of a single-band image *f* can be calculated as follows:

(12) $$\mu =\frac{1}{MN}\sum_{x=1}^{M}\sum_{y=1}^{N}f(x,y)$$

where, *M* and *N* are the row and column numbers, respectively, of image *f*, and *f*(*x*, *y*) is the grey value of the pixel at position (*x*, *y*).

(2) Standard deviation

Standard deviation (SD) reflects the discrete condition of the grey level relative to the grey mean. It can be used to evaluate image-contrast size [37]. Its formula is:

(13) $$\sigma =\sqrt{\frac{\sum_{i=1}^{M}\sum_{j=1}^{N}\left(Z(x_{i},y_{i})-z\right)}{M\times N}}$$

If the SD is small, the image contrast is small. Because the contrast is not big, the tone is single and even, so not too much information is available from the image. The larger the SD, the more dispersed the grey level distribution. The contrast of the image is high, so more information can be obtained from the image.

(3) Entropy

In 1948, Shannon, the founder of modern information theory, defined the mean amount of information as entropy [38]. The formula is:

(14) $$E=-\sum_{i=0}^{255}p_{i}\log_{2}p_{i}$$

where, *p_i_* is the probability of *i* within the range of [0, 255]. Entropy is an important index to measure the richness of image information: it reflects how much information the image carries. A greater entropy of the fused image with respect to that of the non-fused images means that the information contained in the fused image is richer and the fusion effect is better.

(4) Average gradient

Average gradient provides a sensitive reflection of the image’s ability to express contrast between small details [39]. Its formula is:

(15) $$G=\frac{1}{MN}\sum_{i=1}^{M}\sum_{j=1}^{N}\sqrt{\left({\left(\frac{\partial f\left(x_{i},y_{i}\right)}{\partial x_{i}}\right)}^{2}+{\left(\frac{\partial f\left(x_{i},y_{i}\right)}{\partial y_{i}}\right)}^{2}/2\right)}$$

In general, the larger the average gradient, the greater the number of image levels and the clearer the image.

(5) Edge acutance value

Edge acutance value (EAV) is an objective measure of sharpness which considers the sensitivity of the human visual system to specific spatial frequencies and the viewing distance of an image [40]. Edge acutance refers to the ability of a photographic system to show a sharp edge between contiguous areas of low and high illuminance. Its formula is:

(16) $$\mathrm{EAV}=\frac{\sum_{x,y}\sum_{i=1}^{8}\left|df/dx\right|}{M\times N}$$

where *df*/*dx* is the grey change rate of edge vector, and *M* and *N* is respectively the rows and columns of an image.

## 3. Results

### 3.1. Comparison Experiments for Selecting the Sensitive Bands

In this paper, the JSKF, principal components analysis (PCA) [41], covariance matrix eigenvalue (CME) and band ratioing [42] were used to comparatively select the bands of HS images of the same ground. We used these four band-selection methods in a band-selection experiment with the 128 bands of HS images obtained by UHD-185. Table 2 (Stage I) and Table 3 (Stage II) give the band numbers or band ratios of selected bands by the four methods.

It can be found that the bands selected by the PCA are concentrated in the latter third of the band range. The selected bands are relatively concentrated. The spectral range of imaging by the UHD-185 camera is concentrated in the same characteristic spectrum. The reflection of the object in the image is essentially uniform, the grey-level distribution of the image is very similar, and a significant amount of redundant information is present. The CME is relatively good and can choose the range of the different spectral bands. However, this method selects too many bands close to the ends of the spectrum. The image quality of these bands is often poor, so they are not the best bands to be exploited. The bands selected by the JSKF are widely distributed and are far from the operating point of the imaging spectrometer. The data collected by this algorithm are highly available. In comparison with the three former methods, band ratioing can acquire the band ratios derived from the two bands with the most and least variations in each segmented sub-image after image segmentation. The environmental effects may be reduced compared with the single spectral band. The spectral distance of two bands for each band ratio, however, are located relatively close in this study.

**Figure 6:**
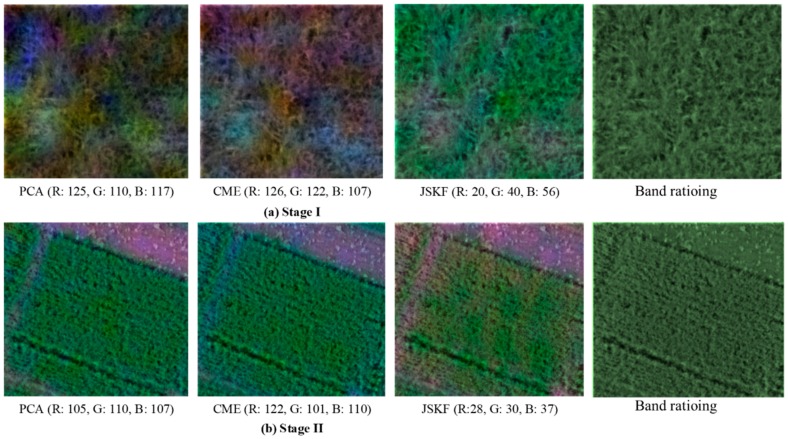
Visual comparison of band selection using the PCA, CME, JSKF and band ratioing.

To further compare the band-selection performance of four methods, we selected two HS images with low- and high-vegetation coverages of wheat at Stage I and Stage II. Three bands or band ratios in HS images were selected by the four methods. Using the first three bands representing the three channel components of R, G, B, false-colour images could be obtained as shown in Figure 6a,b. It can be visually found that the JSKF-based false-colour images have most the obvious colour contrast. Conversely, the colour of false-colour images derived from the band ratioing are generally equalised. Then we used the entropy, SD, and EAVto evaluate the quality of the image after band selection in terms of three aspects. In this way, we can better evaluate how different band-selection methods affect the image. The comparison results are presented in Table 4. We can find that the JSKF performs best for all the evaluation indicators at two growth stages in comparison with the other methods. Band ratioing occupies the second place and CME generally shows the worst performance.

From the above test we can draw a conclusion: the proposed JSKF can overcome the shortcomings induced by the transform-based dimension-reduction method and prevent the original spectral information from being lost. The performance of the proposed method has been also validated by several experiments. The experimental results show that the proposed algorithm can reduce the dimensions of hyperspectral images with little information loss by adaptively selecting the band images. The contour of the image is more prominent, and the spatial information of the target is clear. The sensitive bands extracted by this method are more representative in the whole wave band, which is helpful to analyze the results of the next image fusion experiment.

**Figure 7:**
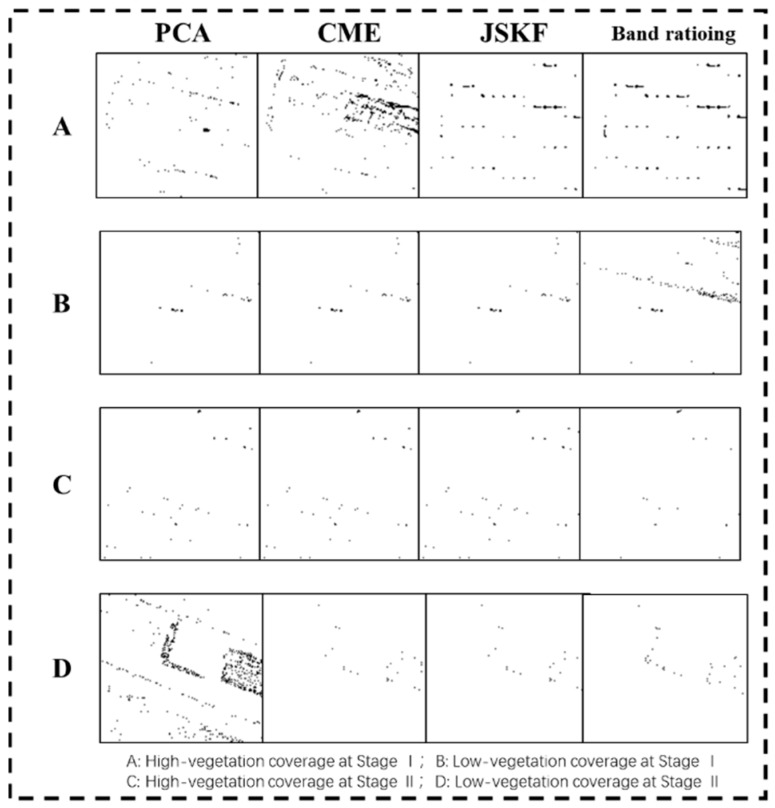
Anomaly detection results with four band-selection methods.

In order to compare the effectiveness of the three band-selection methods, kernel RX (KRX) was used to accomplish the anomaly detection process. To facilitate the analysis of test results, the gray image of the detection results was segmented into binary image by thresholding. Then the morphological filtering method was used to filter out the connected regions with the pixel values greater than 100 to filter out the large area of false alarm area (Figure 7). We can draw a conclusion that the detection effect under the JSKF is better than the other three comparison methods. It enables targets to be easily detected with fewer alarms.

**Figure 8:**
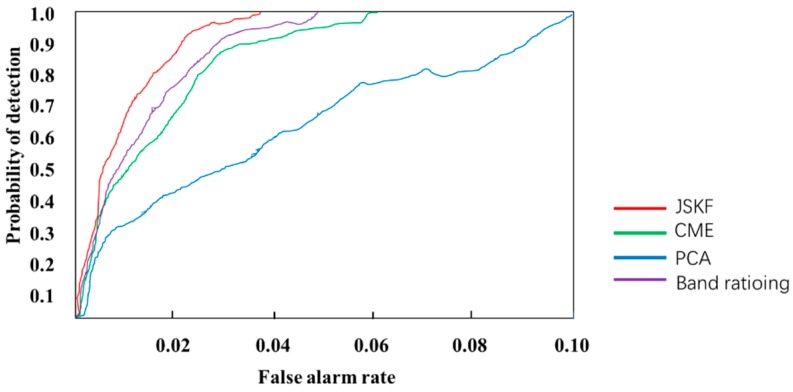
Comparison of ROC curves of anomaly detection results.

Moreover, the receiver operating characteristics (ROC) curve was also used to quantitatively evaluate the test results (Figure 8). It shows that the ROC curve derived from the JSKF method is located above the curves generated by the CME, PCA and band ratioing, which indicates that the anomaly detection results with the JSKF method is better than the other three band-selection methods.

### 3.2. Experimental Fusion Results of HS and PAN Images

To realize the pixel-level fusion of HS images and PAN images, we also have to perform the image registration for the original HS images and PAN images and realize the one-to-one correspondence of the objects in the images.

**Figure 9:**
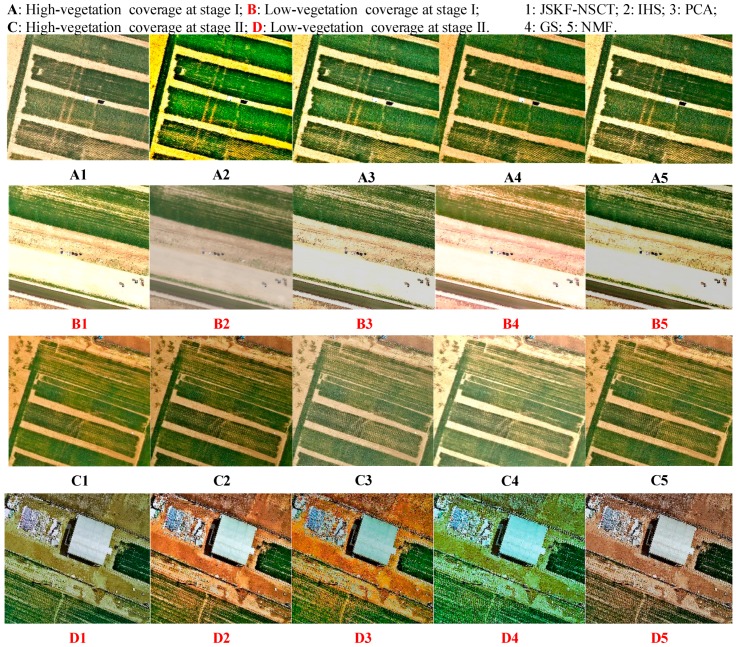
Comparison of fusion results using five different methods.

We used the proposed NSCT-based fusion algorithm to fuse the three bands of HS images based on the above JSKF band-selection rule and PAN images. These image data include remotely sensed HS images and corresponding high-resolution digital images of the same plot of wheat at two growth stages. We used this method and four other traditional methods to fuse these images at each growth stage, two plots with low- and high-vegetation coverages were selected as the control experiments (Figure 9). A and B represent the images with the two coverages at Stage I. Similarly, C and D represent the images at Stage II. Figure 9A1–D1 present the fused images generated by the proposed algorithm. Figure 9A2–D2 are generated by the IHS method, Figure 9A3–D3 by the PCA method, Figure 9A4–D4 by the GS method, and Figure 9A5–D5 by the non-negative matrix factorization (NMF) method.

## 4. Discussion

### 4.1. Evaluation of Fusion Effects

In essence, the quality of fused images consists of three factors: detectability, resolution, and scalability. The detectability of an image indicates the sensitivity of the image to a spectral section. The resolution of the image indicates the ability of the image to provide sufficient contrast for the visual distinction of two small objects. The scalability of an image indicates the ability of an image to correctly restore the shape of the original scene. The detectability and resolution of images are collectively referred to as the quality of image formation, and the image scalability is called the geometric quality of an image. The evaluation of geometric quality is relatively simple and intuitive: it represents the difference between the image points and the corresponding ideal image points in the geometric positions of the remote sensors. The evaluation of image texture quality is complex and difficult and includes not only the image expression level but also the influence of the microstructure on the image quality. It is also related to the requirements of the image user. In many cases, the image quality of a given image is often evaluated differently by different users. At present, the methods of evaluating image fusion are divided into two types: spatial analysis and spectral analysis:

(1) Spatial analysis

Upon comparing the fused images with the original HS image, we see that all fused images have better spatial qualities. Different ground objects can be clearly identified and the image outlines are also clearer. In terms of brightness, the fusion algorithm proposed in this paper has higher brightness relative to several traditional algorithms. This increases the target recognition accuracy under the same terrain conditions. In terms of clarity, the method used in this paper provides better results than other methods, which makes it easier to obtain information from images. At the same time, these methods perform differently in the spectral aspect [43]. However, this kind of evaluation may be too reliant on humans and thus it is necessary to employ quantitative measures. The result of the fused images can be evaluated in terms of two aspects: the spatial resolution of each spectral band image and the spectral quality of each spectrum in a single pixel [44]. Four typical metrics are introduced below as shown in Table 5, Table 6, Table 7 and Table 8. We can find that the values of four indicators of our proposed algorithm are best than the four typical fusion methods.

In Table 5, Table 6, Table 7 and Table 8, some image-based indices are used to test the information and sharp change rate. Moreover, another four indices including correlation coefficient (CC), erreur relative globale adimensionnelle de synthese (ERGAS), root mean square error (RMSE) and bias are also introduced to evaluate the fusion effect (Table 9). Ideally, the values of bias and RMSE are 0. Smaller value shows that more spectral information can be maintained in the fusion results. Similarly, the ideal value of CC is 1. When the value of ERGAS is greater than 3, it shows the poor quality of the fused image, conversely, the fused image is good. As shown in Table 5, Table 6, Table 7, Table 8 and Table 9, we can draw the conclusion that the results obtained by JSKF-NSCT are superior to other fusion methods. Meanwhile, compared with the original images, the fusion images perform better in color details and spectral characteristics.

**Figure 10:**
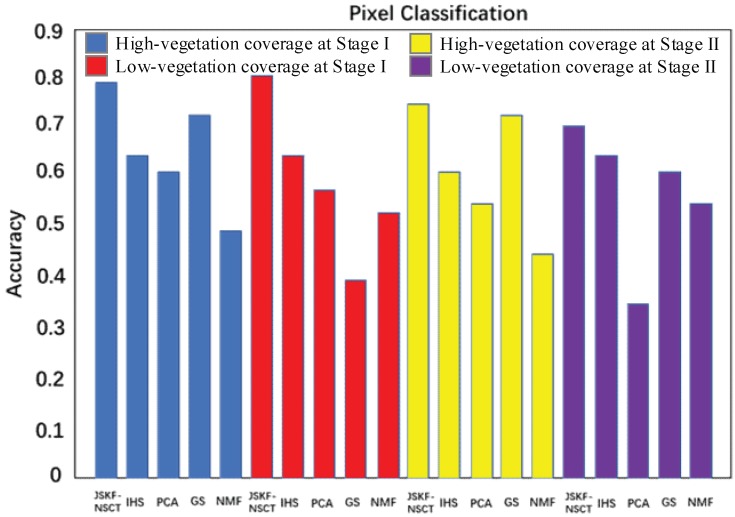
Pixel clustering accuracy using the five fusion algorithms.

In addition to objectively evaluate the methods discussed in Section 2.2 and Section 3.1, pixel clustering was also used to accomplish the further validation of our results below. It is believed that an algorithm with higher CC, RMSE or ERGAS can also perform well in pixel clustering analysis. Figure 10 shows the clustering results from five methods based on the cluster centres extracted from ground truth fusion images, respectively. Here, the K-means algorithm was used to locate the cluster centres. It can be seen that in comparison with the other four methods, the JSKF-NSCT method can achieve the highest clustering accuracy.

(2) Spectral analysis

In this paper, the high-resolution PAN image and HS images of two growing periods are processed by the JSKF-NSCT fusion algorithm. The normalised difference vegetation index (NDVI) of the fused image was calculated and modelled by using the leaf area index (LAI) of the measured plots and the chlorophyll of the corresponding region [45]. Subsequently, the same operation was performed with four other traditional methods. Next, the modelling process was carried out and the coefficients of determination (*R*^2^) obtained by the methods were compared [46,47]. The closer *R*^2^ is approaching 1, the better the fusion image reflects the real ground vegetation coverage, which means the fusion result is better. The results are compared in Table 10 and Table 11.

### 4.2. Characteristics and Drawbacks of Traditional Methods

Generally speaking, the IHS transform fusion method improves the texture features of a target image. At the same time, the fusion results maintain the characteristics of HS images in terms of hue, saturation, and so on [48]. However, the spectral information suffers certain losses. Moreover, the IHS fusion method can only fuse three bands. In addition, the IHS transform distorts the spectral features of the original multispectral image, resulting in spectral degradation [49].

PCA is a widely used method of fusion and focuses on the fusion transform over three-band images [50]. The main advantage of the fusion algorithm is that the spectral characteristics of the fused images remain better, especially in the case of too many bands. The disadvantage is that, because the eigenvalues and eigenvectors of the autocorrelation matrix are to be calculated, the computational complexity is very large and the real-time performance is poor [51]. In addition, the principal components of the PCA transform lose their original physical characteristics, and the method is very sensitive to the selection of the fusion region.

NMF method [52] can better extract and describe the local-feature information of the image, so as to achieve better expression of the image by simulating the human brain’s cognition of the image information. It is a multivariate analysis method and is essentially a matrix decomposition and projection technique. Its basic principles can be described as follows:

For any arbitrary nonnegative matrix $V=\left[\nu_{1},\nu_{2},\cdots \nu_{N}\right]$, the NMF method requires finding a non-negative *M* × *L* basis matrix *W* and an *L* × *N* coefficient matrix *H*. These matrices must satisfy the condition:

(17) $$V_{M\times N}\approx W_{M\times L}\cdot H_{L\times N}$$

It can integrate the dominant regions of different remotely sensed images and strengthen the regional characteristics. It thus improves the result of the fused image. However, the NMF method involves significant computational complexity, making it inefficient for dealing with remotely sensed images containing large amounts of information.

The GS algorithm [53] is a multidimensional linear transformation that is often used in statistics. The GS transform is used to process the multi-dimensional data of HS images so that redundant information can be eliminated. The basic step of the GS fusion method is to first produce the first component by spectral resampling, thereby converting HS images into orthogonal spaces. Finally, the fused image is obtained by an inverse GS transformation. However, the high spatial resolution image differs significantly from the harmonic phase and pixel value. After the GS transform, a big difference remains between the grey values of image pixels and the remaining components of harmonics.

Until recently, the multi-resolution decomposition-based algorithms have been widely used in the field of multisource image fusion and have effectively overcome spectrum distortion. Wavelet transformation provides great time-frequency analytical features and is the focus of multisource image fusion [54]. The above methods are made up of the tensor product of two one-dimensional wavelets, solving the problem of lack of shift invariance, which traditional wavelets cannot do. As they lack anisotropy, these methods fail to express direction-distinguished texture and edges sparsely.

(1) Advantages of proposed method and analysis of experimental results

Compared with the traditional remote-sensing image-fusion algorithm, NSCT inherits the advantages of the above algorithms and also benefits from translation invariance, which can greatly reduce the influence of registration error on the fusion performance. At the same time, each subband image obtained by NSCT decomposition has the same size as the original image, so it is easy to find the corresponding relationship among the subbands, which is beneficial for the development of the fusion rules.

**Figure 11:**
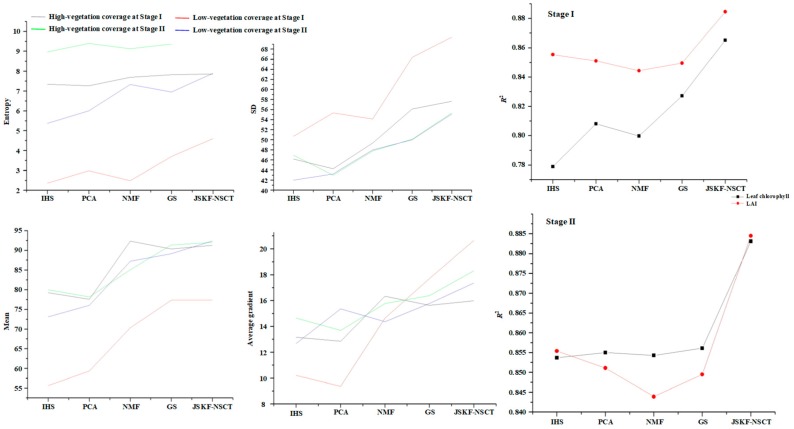
Comparison of the fusion results among our proposed algorithm and other fusion methods.

From the contrast experiments described above, we can compare the results of the proposed algorithm with those of several traditional methods. The fusion results obtained by the method proposed herein and embodied by the set of several indices are significantly improved. Figure 11 compares the results of the various fusion methods.

They show that, for multiple evaluation indicators, at different growth stages and different vegetation coverages, the fusion results obtained by using the method proposed herein are significantly improved compared with the results of the traditional fusion methods. This suggests that the band selection and fusion algorithm proposed in this paper are robust. The improvements are listed in Table 12, Table 13, Table 14 and Table 15.

The above results show that, after processing by the proposed band-selection method and fusion algorithm, the resulted target image contains more information and clearer edges. Image fusion with different models and numerical tests were conducted in our experiments [55], and the four experiments described above indicate that the proposed method has notable superiority in image fusion performance over the four other techniques examined and has better robustness and timeliness. We observed that images based on our proposed method offer the best visual effect and those based on PCA are the worst. In addition to visual inspection, quantitative analysis is also conducted to verify the validity of our algorithm from the viewpoint of entropy, SD, average gradient, and mean. The values of these metrics indicate that the experiments achieve the desired objective.

(2) Suitability evaluation of proposed method

Note that this study has examined only several images of two growing stages in a single year. Due to partial absence of the original hyperspectral images, we have not been able to implement a full analysis of the images of the entire crop-reproduction period. Moreover, the analysis of crop variety is singular; whether this method has the same effect on other different varieties of crop images remains unknown. However, these problems could be solved if we get consecutive years of image data for different crops.

### 4.3. Computationally Efficiency with Three Fusion Methods

Speed is critical for image fusion in the field condition. In this paper, the average running time is used to evaluate the efficiency (Table 16). The running platform of all methods is a Windows 10 PC with 4-core Intel Corn I5 processor (3.60 GHz) with 12 GB RAM. We can see that the average running time of JSKF-NSCT, PCS and GS are 15.4 s, 17.6 s and 14.4 s, respectively. Although the JSKF-NSCT method does not reach the shortest running time, it achieves a more efficient processing speed while taking account into fusion accuracy. The mean values of time-consuming are 7.4 s (band selection), 2.5 s (selection of fusing coefficients) and 5.5 s (image fusion), respectively. In addition, the key to promote the computationally efficiency is to improve the selection rules of fusing coefficients and maybe the interval optimization algorithm is a good alternative. It can be imaged that the running time will be shortened in the future, and the proposed fusion method are more suitable for handheld operations in the field.

## 5. Conclusions

In this paper, we focus on PAN images, which have high spatial resolution but lack spectral information, and HS images, which have high spectral resolution and rich spectral information but lack spatial resolution. To solve this problem, we propose a fusion algorithm for UAV HS and PAN digital images combining the JSKF and NSCT. First, we use the JSKF model to extract the sensitive bands from HS images, and then we fuse the extracted sensitive HS image bands and the corresponding PAN images using the NSCT image-fusion algorithm. At the same time, traditional PCA, IHS, NMF and GS methods are comparatively used to process the same object. The results of the five algorithms are evaluated using the image indices and agronomy indices. The experimental results show that the proposed fusion method can significantly improve the spatial resolution of HS images and also maintain rich spectral characteristics. The fused image contains more information than the original images. Furthermore, the image’s detail contrast, texture, and resolution are greatly improved, and the quality of the fused image is better. In addition, the JSKF-NSCT fusion method can also maintain a satisfactory processing speed while taking account into fusion accuracy.

## Figures and Tables

**Table 1:** Primary steps for performing the NSCT based fusion of HS and PAN images.

Operation Procedures: NSCT Based Fusion Method
(1) An IHS transform is applied to the false-colour image derived from the JSKF model to obtain the three components of *I*, *H*, and *S*. (2) The *I* components of the HS and PAN images are extracted using the NSCT to obtain the frequency coefficients. (3) Weighted fusion of the low-frequency coefficients is applied and the high-frequency coefficients are selected by using the correlation between the centre pixel and its surrounding pixels. (4) NSCT is used to reconstruct the fusion coefficients. (5) The inverse IHS transform is used to obtain the final fusion result.

**Table 2:** Comparison of band indices selected by four methods at Stage I.

Band-Selection Method	Selected Bands or Band Ratios
PCA	125, 110, 117, 120, 98, 101, 112, 125, 99, 105
CME	126, 122, 107, 6, 98, 5, 9, 19, 99, 103
JSKF	20, 40, 56, 50, 105, 114, 120, 100, 99, 102
Band ratioing	117/125, 105/120, 101/99

**Table 3:** Comparison of band indices selected by four methods at Stage II.

Band-Selection Method	Selected Bands or Band Ratios
PCA	105, 110, 107, 90, 101, 119, 112, 125, 98, 108
CME	122, 101, 100, 23, 8, 66, 7, 102, 123, 90, 126
JSKF	28, 30, 37, 59, 78, 90, 100, 108, 96, 121
Band ratioing	107/105, 112/98, 90/108

**Table 4:** Quantitative evaluation of band selection.

Indicator	Stage I				Stage II			
	PCA	CME	JSKF	Band Ratioing	PCA	CME	JSKF	Band Ratioing
Entropy	5.23	4.96	7.98	6.94	7.21	7.13	8.97	7.12
SD	8.26	7.63	10.59	9.21	10.36	9.58	12.79	10.87
EAV	2.26	2.10	3.36	3.11	3.54	3.62	4.38	3.97

**Table 5:** Comparison of evaluation for five fusion methods for experiment A.

Method	Entropy	SD	Average Gradient	Mean
IHS	7.347	46.174	13.164	79.268
PCA	7.268	44.235	12.859	77.569
NMF	7.695	49.356	16.338	91.265
GS	7.826	56.125	15.632	90.365
JSKF-NSCT	7.859	57.673	15.997	92.365

**Table 6:** Comparison of evaluation for five fusion methods for experiment B.

Method	Entropy	SD	Average Gradient	Mean
IHS	2.365	50.698	10.205	55.695
PCA	2.985	55.369	9.368	59.365
NMF	2.489	54.127	14.639	70.359
GS	3.698	66.358	17.698	77.369
JSKF-NSCT	4.601	70.369	20.656	80.456

**Table 7:** Comparison of evaluation for five fusion methods for experiment C.

Method	Entropy	SD	Average Gradient	Mean
IHS	8.965	46.889	14.635	80.006
PCA	9.397	42.976	13.698	78.192
NMF	9.125	47.787	15.779	85.127
GS	9.368	50.129	16.383	91.368
JSKF-NSCT	9.975	55.363	18.309	92.001

**Table 8:** Comparison of evaluation for five fusion methods for experiment D.

Method	Entropy	SD	Average Gradient	Mean
IHS	5.369	42.012	12.687	73.127
PCA	6.002	43.226	15.368	76.065
NMF	7.331	47.997	14.365	87.245
GS	6.957	50.012	15.778	89.148
JSKF-NSCT	7.897	55.147	17.366	92.386

**Table 9:** Comparison of another four evaluation indices for five fusion methods.

Method	CC	RMSE	ERGAS	Bias
IHS	0.59	3.24	3.7	0.07
PCA	0.62	4.21	2.6	0.48
NMF	0.67	4.62	4.3	0.26
GS	0.67	5.97	3.1	0.05
JSKF-NSCT	0.69	3.17	2.7	0.03

**Table 10:** Results of comparison at Stage I.

Method	Leaf Chlorophyll		LAI	
	Linear Formula	*R* ^2^	Linear Formula	*R* ^2^
IHS	*y* = −0.002*x* + 2.7574	0.7790	*y* = 0.2802*x* + 7.2984	0.8554
PCA	*y* = −0.0022*x* + 2.7844	0.8081	*y* = 0.2615*x* + 7.0921	0.8511
NMF	*y* = −0.0022*x* + 2.7869	0.7998	*y* = 0.2568*x* + 7.1717	0.8444
GS	*y* = −0.0024*x* + 2.8128	0.8272	*y* = 0.2488*x* + 6.8835	0.8496
JSKF-NSCT	*y* = −0.003*x* + 2.8826	0.8653	*y* = 0.2328*x* + 5.3181	0.8846

**Table 11:** Results of comparison at Stage II.

Method	Leaf Chlorophyll		LAI	
	Linear Formula	*R* ^2^	Linear Formula	*R* ^2^
IHS	*y* = 0.0005*x* + 0.0009	0.8537	*y* = 0.3098*x* + 8.0463	0.8554
PCA	*y* = 0.0005*x* + 0.0091	0.8550	*y* = 0.2882*x* + 7.795	0.8511
NMF	*y* = 0.0005*x* + 0.0092	0.8543	*y* = 0.2827*x* + 7.8869	0.8439
GS	*y* = 0.0005*x* + 0.8561	0.8561	*y* = 0.2729*x* + 7.5321	0.8495
JSKF-NSCT	*y* = 0.0004*x* + 0.008	0.8831	*y* = 0.2445*x* + 5.5826	0.8845

**Table 12:** Percent improvement in entropy.

	Stage I		Stage II	
	High-Vegetation Coverage	Low-Vegetation Coverage	High-Vegetation Coverage	Low-Vegetation Coverage
IHS	6.96%	94.5%	11.2%	47.1%
PCA	8.13%	54.1%	6.15%	31.6%
NMF	2.13%	84.8%	9.31%	7.72%
GS	0.42%	24.4%	6.47%	13.5%

**Table 13:** Percent improvement in SD.

	Stage I		Stage II	
	High-Vegetation Coverage	Low-Vegetation Coverage	High-Vegetation Coverage	Low-Vegetation Coverage
IHS	24.8%	38.8%	18.1%	31.2%
PCA	30.3%	27.1%	28.8%	27.5%
NMF	16.8%	30.0%	15.8%	14.9%
GS	2.75%	6.04%	10.4%	10.2%

**Table 14:** Percent improvement in average gradient.

	Stage I		Stage II	
	High-Vegetation Coverage	Low-Vegetation Coverage	High-Vegetation Coverage	Low-Vegetation Coverage
IHS	21.5%	102.4%	25.1%	36.8%
PCA	24.4%	120.4%	33.6%	13.0%
NMF	−2.08%	41.1%	16.0%	20.8%
GS	2.33%	16.7%	11.7%	10.1%

**Table 15:** Percent improvement in mean.

	Stage I		Stage II	
	High-Vegetation Coverage	Low-Vegetation Coverage	High-Vegetation Coverage	Low-Vegetation Coverage
IHS	15.1%	44.4%	14.9%	26.3%
PCA	17.6%	35.5%	17.6%	21.4%
NMF	−1.19%	14.3%	8.07%	5.89%
GS	0.99%	3.98%	0.69%	3.63%

**Table 16:** Test and comparison of computationally efficiency.

Method	Average Running Time (s)
JSKF-NSCT	15.4
PCA	17.6
GS	14.4
